# Supplementary material for: Centralized scientific communities are less likely to generate replicable results
Source: eLife. 2019 Jul 2;8:e43094. doi: 10.7554/eLife.43094 (PMC6606034; doi:10.7554/eLife.43094)
Supplement: Supplementary file 3. [file elife-43094-supp3.docx]

Danchev, Rzhetsky and Evans. 2019. Centralized scientific communities are less likely to generate replicable results. *eLife* **8**:e43094 DOI: doi.org/10.7554/eLife.43094

***Supplementary file 3***

**Table S1. Logistic regression models with claim replication *R* [Replicated = 1, Non-replicated = 0] as response variable and predictors modelled independently.**

| Variable | *B* | *SE* | *Exp(B)* | *95% CI* | *P* |
| --- | --- | --- | --- | --- | --- |
|  |  |  |  |  |  |
| **Intercept** | –0.762 | 0.195 | 0.47 | [0.318, 0.684] | <0.001 |
| **Support in literature** | 3.144 | 0.479 | 23.2 | [9.078, 59.287] | <0.001 |
| **Intercept** | 0.414 | 0.046 | 1.513 | [1.384, 1.654] | <0.001 |
| **Journal prominence** | 2.379 | 0.564 | 10.795 | [3.571, 32.638] | <0.001 |
| **Intercept** | ­–1.053 | 0.193 | 0.349 | [0.239, 0.509] | <0.001 |
| **Social independence** | 1.843 | 0.224 | 6.312 | [4.069, 9.793] | <0.001 |
| **Intercept** | –1.004 | 0.255 | 0.366 | [0.222, 0.604] | <0.001 |
| **Methodological independence** | 1.841 | 0.308 | 6.301 | [3.444, 11.527] | <0.001 |
| **Intercept** | –1.14 | 0.376 | 0.32 | [0.153, 0.668] | 0.002 |
| **Prior knowledge independence** | 1.71 | 0.388 | 5.53 | [2.584, 11.835] | <0.001 |
| **Intercept** | 0.585 | 0.065 | 1.794 | [1.579, 2.039] | <0.001 |
| **Variability in LINCS L1000** | -0.444 | 0.264 | 0.641 | [0.382, 1.077] | 0.093 |
| **Intercept** | 0.8 | 0.062 | 2.224 | [1.972, 2.51] | <0.001 |
| **Centralization** | –1.018 | 0.149 | 0.361 | [0.27, 0.484] | <0.001 |

**Logistic regression models with claim replication *R* [Replicated = 1, Non-replicated = 0] as response variable and predictors modelled independently.** Predictors are rescaled $\frac{x_{i}-min(x)}{\max\left( x \right)-min(x)}$ for comparability. *N* = 2,493 claims (*N* = 2,491 in the Journal eigenfactor model).
