# Supplementary material for: Centralized scientific communities are less likely to generate replicable results
Source: eLife. 2019 Jul 2;8:e43094. doi: 10.7554/eLife.43094 (PMC6606034; doi:10.7554/eLife.43094)
Supplement: Supplementary file 4. [file elife-43094-supp4.docx]

Danchev, Rzhetsky and Evans. 2019. Centralized scientific communities are less likely to generate replicable results. *eLife* **8**:e43094 DOI: doi.org/10.7554/eLife.43094

***Supplementary file 4***

**Table S2. Logistic regression models with claim replication *R* [Replicated = 1, Non-replicated = 0] as response variable and predictors modelled simultaneously.**

| Variable | *B* | *SE* | *Exp(B)* | *95% CI* | *P* |
| --- | --- | --- | --- | --- | --- |
|  |  |  |  |  |  |
| **Intercept** | –0.693 | 0.352 | 0.5 | [0.251, 0.996] | 0.049 |
| **Support in literature** | 2.729 | 0.595 | 15.32 | [4.772, 49.153] | <0.001 |
| **Journal prominence** | 1.019 | 0.563 | 2.769 | [0.918, 8.355] | 0.071 |
| **Social independence** | 0.632 | 0.486 | 1.881 | [0.725, 4.877] | 0.194 |
| **Methodological independence** | –0.183 | 0.585 | 0.833 | [0.265, 2.621] | 0.754 |
| **Prior knowledge independence** | –0.029 | 0.769 | 0.971 | [0.215, 4.383] | 0.970 |
| **Variability in LINCS L1000** | –0.41 | 0.271 | 0.664 | [0.391, 1.128] | 0.130 |
| **Centralization** | –0.734 | 0.268 | 0.48 | [0.284, 0.812] | 0.006 |

**Logistic regression models with claim replication *R* [Replicated = 1, Non-replicated = 0] as response variable and predictors modelled simultaneously.** Predictors are rescaled $\frac{x_{i}-min(x)}{\max\left( x \right)-min(x)}$ for comparability. *N* = 2,491 claims.
